# A single digital droplet PCR assay to detect multiple *KIT* exon 11 mutations in tumor and plasma from patients with gastrointestinal stromal tumors

**DOI:** 10.18632/oncotarget.24493

**Published:** 2018-02-14

**Authors:** Pieter A. Boonstra, Arja ter Elst, Marco Tibbesma, Lisette J. Bosman, Ron Mathijssen, Florence Atrafi, Frits van Coevorden, Neeltje Steeghs, Sheima Farag, Hans Gelderblom, Winette T.A. van der Graaf, Ingrid M.E. Desar, Jacqueline Maier, Jelle Overbosch, Albert J.H. Suurmeijer, Jourik Gietema, Ed Schuuring, Anna K.L. Reyners

**Affiliations:** ^1^ University of Groningen, University Medical Center Groningen, Department of Medical Oncology, Groningen 9713 GZ, The Netherlands; ^2^ University of Groningen, University Medical Center Groningen, Department of Pathology, Groningen 9713 GZ, The Netherlands; ^3^ Department of Medical Oncology, Erasmus University Medical Center Rotterdam, Rotterdam 3015 CE, The Netherlands; ^4^ Antoni van Leeuwenhoek, Netherlands Cancer Institute, Department of Surgery, Amsterdam 1066 CX, The Netherlands; ^5^ Antoni van Leeuwenhoek, Netherlands Cancer Institute, Department of Medical Oncology, Amsterdam 1066 CX, The Netherlands; ^6^ Leiden University Medical Center, Department of Medical Oncology, Leiden 2300 RC, The Netherlands; ^7^ Radboud University Medical Center, Department of Medical Oncology, Nijmegen 6500 HB, The Netherlands; ^8^ University of Leipzig, Center for Internal Medicine, Department of Hematology/Oncology, Leipzig 04103, Germany; ^9^ University of Groningen, University Medical Center Groningen, Department of Radiology, Groningen 9713 GZ, The Netherlands

**Keywords:** GIST, cell-free circulating DNA, single assay, plasma, digital droplet PCR

## Abstract

**Background:**

Gastrointestinal stromal tumors (GISTs) are characterized by oncogenic *KIT* mutations that cluster in two exon 11 hotspots. The aim of this study was to develop a single, sensitive, quantitative digital droplet PCR (ddPCR) assay for the detection of common exon 11 mutations in both GIST tumor tissue and in circulating tumor DNA (ctDNA) isolated from GIST patients’ plasma.

**Methods:**

A ddPCR assay was designed using two probes that cover both hotspots. Available archival FFPE tumor tissue from 27 consecutive patients with known KIT exon 11 mutations and 9 randomly selected patients without exon 11 mutations were tested. Plasma samples were prospectively collected in a multicenter bio-databank from December 2014. ctDNA was analyzed of 22 patients with an exon 11 mutation and a baseline plasma sample.

**Results:**

The ddPCR assay detected the exon 11 mutation in 21 of 22 tumors with exon 11 mutations covered by the assay. Mutations in ctDNA were detected at baseline in 13 of 14 metastasized patients, but in only 1 of 8 patients with localized disease. In serial plasma samples from 11 patients with metastasized GIST, a decrease in mutant droplets was detected during treatment. According to RECIST 1.1, 10 patients had radiological treatment response and one patient stable disease.

**Conclusion:**

A single ddPCR assay for the detection of multiple exon 11 mutations in ctDNA is a feasible, promising tool for monitoring treatment response in patients with metastasized GIST and should be further evaluated in a larger cohort.

## INTRODUCTION

Gastrointestinal stromal tumors (GISTs) are rare malignancies of the gastrointestinal tract [1]. GIST is known to have driver single nucleotide variants, deletions and insertions (further referred to as mutations) in genes encoding the tyrosine kinase receptors *KIT* and *PDGFRα.* These occur in respectively 80% and 10% of GIST patients [2, 3]. In untreated GIST patients, the most frequent mutations are in *KIT* exon 11 (70%) coding for the juxta-membrane domain, and *KIT* exon 9 (10% of patients), coding for the extracellular domain of the receptor [4]. Around 80% of the mutations in *KIT* exon 11 cluster in two hotspot regions of approximately 25 base pairs within a 100 base-pair range of each other [5].

Therapy consists of surgery and/or treatment with one of several selective tyrosine kinase inhibitors (TKIs). Adjuvant treatment with a TKI is based on Miettinen’s risk classification which takes the size of the tumor, location and mitotic index into account [6]. Independently of the Miettinen classification, there is also a difference in recurrence risk between GISTs with different *KIT* and *PDGFRα* mutations [7]. First line treatment for locally advanced or metastatic GISTs consists of imatinib (400 mg daily), a selective inhibitor of the *KIT* tyrosine kinase [8]. Almost all patients with exon 11 mutated GIST respond to imatinib treatment, whereas exon 9 mutated GISTs have lower response rates. A large meta-analysis of 1,640 patients showed that bi-daily imatinib 400 mg is more effective than once daily dosing in *KIT* exon 9 mutated patients [9]. Resistance to imatinib treatment is usually the result of one or multiple secondary mutations that develop during treatment [10, 11].

Second and third line treatment, with respectively sunitinib and regorafenib, also showed differential response rates that correlated with the primary mutational status of the tumor [12–14].

Molecular diagnostic testing of relevant predictive biomarkers, including *KIT* and *PDGFRα,* is becoming routine practice in clinical decision-making. Mutation detection is routinely performed on pre-treatment tumor biopsies or resection specimens. For the detection of mutations a variety of methods are used, including as Sanger sequencing, pyro-sequencing, next generation sequencing (NGS) and high-resolution-melting (HRM) analysis with reflex sequencing [15]. These techniques are expensive, time consuming and require sufficient amounts of DNA (>100 ng) and a sufficient percentage of neoplastic cells (>5–20%). In some cases, no representative tumor material is available for molecular testing. Alternative methods for mutation detection, ideally also allowing serial non-invasive measurements, are urgently needed.

Interestingly, recent advantages in molecular pathology enable the detection of tumor specific mutations in circulating tumor DNA (ctDNA) extracted from blood plasma [16]. CtDNA can be used to define targets for selective therapy in both untreated and TKI-resistant non-small-cell-lung-cancer (NSCLC) tumors [17, 18]. The detection of mutations in ctDNA as a predictive biomarker has been reported in both metastatic breast cancer (MBC) [19] and metastatic colorectal cancer (CRC) [20]. Finally, mutation testing in ctDNA allows monitoring of TKI treatment response, where an increase in mutations could predict recurrence or disease progression [18]. Sporadic reports describe the use of ctDNA to detect mutations in GIST patients [21–24].

ctDNA is present in low amounts in plasma within a much more abundant background of non-tumor DNA (wild type) and is varying based on tumor type [25]. Highly analytical sensitive methods are used to detect ctDNA in plasma, these include BEAMing [26] and digital droplet PCR (ddPCR) [26, 27]. Both BEAMing and ddPCR assays require the use of a separate assay for each tumor specific mutation. In general practice, based on the mutation detected in the tumor sample, a unique assay for the specific mutation is designed. Recently, the use of a single ddPCR assay to simultaneously detect various *EGFR*-exon 19 deletions in the plasma of NSCLC patients was reported [28].

Given the long disease course of GIST patients and the multiple therapeutic options depending on mutational status of the tumor, a non-invasive test that can easily assess the presence of mutations is especially interesting for this patient group. Therefore, the aim of this exploratory study was to develop a ddPCR assay to detect most common exon 11 *KIT* mutations in. For the validation of this drop-off ddPCR assay we tested 36 formalin fixed paraffin embedded (FFPE) pre-treatment biopsies of patients with GIST previously tested for mutations using sequencing. To investigate the utility of this assay for detecting exon 11 mutations in ctDNA, plasma from 22 GIST patients was analyzed at baseline and at various time points during TKI treatment.

## RESULTS

### Mutations in *KIT* exon 11 in GIST FFPE tumor tissue detected using the ddPCR drop-off assay

**Figure 1:**
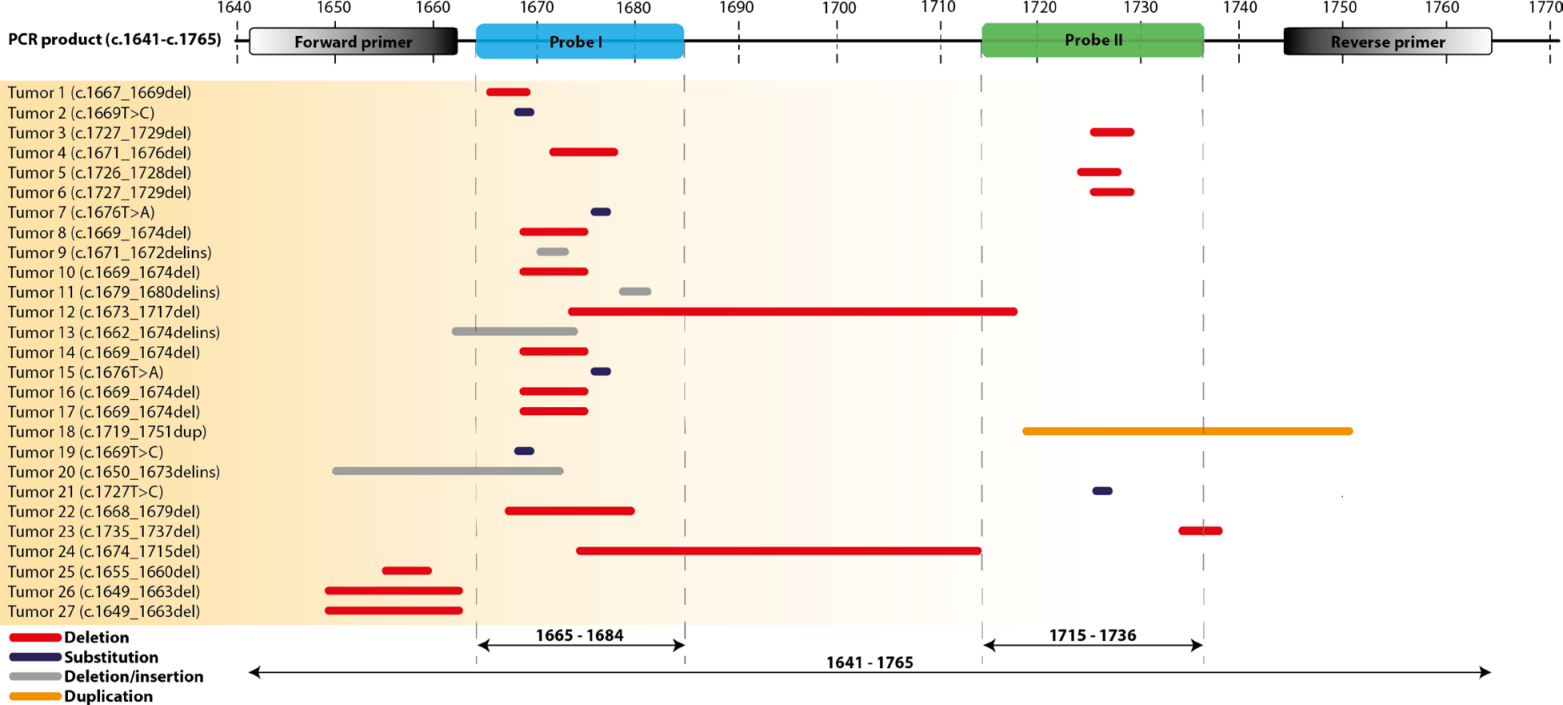
Location of KIT exon 11 mutations in GIST tumour samples as tested with Sanger sequencing or NGS The mutations are displayed relative to the actual position of the Forward and Reverse primers, the two probes (I and II) and PCR product. Type of mutations: red = deletion, grey = substitution, blue = deletion/insertion, orange = duplication.

27 tumors with *KIT* exon 11 mutations were included, 17 tumors had a deletion, 1 a duplication, 4 a deletion/insertion and 5 had single nucleotide variants (SNV) as previously identified by sanger sequencing or NGS. Seventeen mutations cluster in hotspot 1, 6 in hotspot 2, one tumor had a deletion affecting both hotspots (sample 12) and in 3 tumors the deletion did not occur within the hotspots (samples 25, 26, 27, Figure 1). Using the drop-off assay, a *KIT* mutation was detected in 21/27 tumors (see examples in Supplementary Figure 1). Tumor 18 had a duplication and was considered negative in the drop-off assay, however a typical pattern of droplet distribution was seen (Supplementary Figure 2). In 4 of the 5 negative tumors (20, 25, 26 and 27) the deletion did not allow annealing of the PCR-primer and therefore a PCR-product could not be generated (true negative tumors). Tumor 21 carried a SNV within the detection range of probe 2 and was the only true false-negative tumor. Of 22 tumors with mutations in *KIT* exon 11 covered by the drop-off assay, 21 tumors were positive resulting in a sensitivity of 95%. Analysis of the allelic frequency of mutant alleles versus wild type alleles of ddPCR corroborated the NGS results (Table 1). As a negative control, 9 tumors without *KIT* exon 11 mutations were analyzed. These consisted of 4 tumors with a *PDGFRα* mutation, 2 with a *KIT* exon 9 mutation and 3 without any *KIT*/*PDGFRα* mutations. All control GIST samples were negative resulting in a specificity of 100% (Supplementary Figure 3).

**Table 1:** Tested patients with drop-off assay in relation to NGS results

	Allelic frequency		Mutation		
Patient	Mutant	Mutant	in probe area	Mutation	
	ddPCR	NGS			
1	44,00%	38%	**1**	KIT Exon 11	c.1667_1669del
2	58,00%	61%	**1**	KIT Exon 11	c.1669T>C
3	53,00%	59%	**2**	KIT Exon 11	c.1727_1729del
4	41,00%	41%	**1**	KIT Exon 11	c.1671_1676del
5	30,00%	27%	**2**	KIT Exon 11	c.1726_1728del
6	34,00%	38%	**2**	KIT Exon 11	c.1727-1729delTTC
7	42,00%	38%	**1**	KIT Exon 11	c.1676T>A
8	45,00%	45%	**1**	KIT Exon 11	c.1669_1674del
9	54,00%	55%	**1**	KIT Exon 11	c.1671_1672delinsTG
10	40,00%	38%	**1**	KIT Exon 11	c.1669_1674del
11	83,00%	80%	**1**	KIT Exon 11	c.1679_1680delinsAG
12^*^	22,00%	28%	**1,2**	KIT Exon 11	c.1673_1717del
13	24,00%	45%	**1**	KIT Exon 11	c.1662_1674delinsGGAAGAA
14	88,00%	91%	**1**	KIT Exon 11	c.1669_1674del
15	35,00%	34%	**1**	KIT Exon 11	c.1676T>A
16	35,70%	57%	**1**	KIT Exon 11	c.1669_1674delTGGAAG
17	22,30%	25%	**1**	KIT Exon 11	c.1669_1674del
18$	0,00%	11%	**2**	KIT Exon 11	c.1719_ 1751dup
19	43,60%	44%	**1**	KIT Exon 11	c.1669T>C
20&	0,00%	79%	**1**	KIT Exon 11	c.1650_1673delinsCCTTCG
21	0,00%	Sanger	**2**	KIT Exon 11	c.1727 T>C
22	95,00%	86%	**1**	KIT Exon 11	c.1668_1679del
23	41,00%	Sanger	**2**	KIT Exon 11	c.1735_1737del
24	47,00%	Sanger	**1**	KIT Exon 11	c.1674_1715del
25&	0,00%	41%	**0**	KIT Exon 11	c.1655_1660delTGTATG
26&	0,00%	56%	**0**	KIT Exon 11	c.1649_1663del
27&	0,00%	44%	**0**	KIT Exon 11	c.1649_1663del

Detection of mutations in *KIT* with the ddPCR assay of pre-treatment tumor samples compared to the mutant allelic frequency as determined with NGS.

^*^despite a deletion partially overlapping with both hotspot areas, a signal was observed with probe 2;

^&^deletion located in the forward primer annealing site precluding amplification of the mutated allele;

^$^duplication considered negative, however a typical pattern of droplet distribution was seen.

For representative examples see Supplementary Figure 1.

### Tumor and treatment characteristics

Plasma samples taken before start of TKI treatment (baseline) of 22 patients with GIST were analyzed. Of these 22 patients, 14 had metastatic disease and 8 localized disease. Four patients with localized disease were planned to start with imatinib 400mg treatment in a neo-adjuvant setting (patient 7, 9, 13 and 17) and four patients underwent primary surgery. Samples of patients with metastatic disease were taken before start of a new line of TKI treatment (Table 2).

**Table 2:** Plasma ctDNA analysis with drop-off ddPCR assay of GIST patients with metastasized disease

Patient	Primary GIST location	Prior treatment	New treatment	Mutation		Fractional abundance
3	Stomach	Imatinib	Sunitinib	KIT exon 11	c.1727_1729del	12,00%
4	Stomach	-	Imatinib	KIT exon 11	c.1671_1676del	0,40%
6	Small bowel	Imatinib Sunitinib	Regorafenib	KIT exon 11	c.1727-1729del	0,00%
11	Small bowel	-	Imatinib	KIT exon 11	c.1679_1680delinsAG	0,10%
14	Stomach	-	Imatinib	KIT exon 11	c.1669_1674del	14,20%
15	Small bowel	-	Imatinib	KIT exon 11	c.1676T>A	1,00%
16	Stomach	-	Imatinib	KIT exon 11	c.1669_1674del	1,40%
39	Small bowel	Imatinib Sunitinib Regorafenib	-	KIT exon 11	c.1676_1684del	3,00%
40	Small bowel	-	Imatinib	KIT exon 11	c.1668_1717delinsACCTT	7,00%
41	Stomach	-	Imatinib	KIT exon 11	c.1671_1715del	8,70%
42	Stomach	Imatinib	Masitinib	KIT exon 11	c.1670_1675del	0,90%
43	Small bowel	-	Imatinib	KIT exon 11	c.1676T>A	0,40%
44	Small bowel	-	Imatinib	KIT exon 11	c.1665_1676del	0,90%
45	Stomach	Imatinib	Sunitinib	KIT exon 11	c.1674_1695del	3,10%

Plasma samples were collected before start of a new line of TKI treatment. Pre-treatment primary tumors of 12 patients tested positive with the drop-off assay (patient 3–16 see Table 1, patient 39–43 data not shown, patient 44 and 45 were not tested due to lack of tumors tissue). The fractional abundance was determined using DNA input representing 4 ml plasma.

### Detection of exon 11 mutations in ctDNA with the drop-off ddPCR assay

*KIT* exon 11 mutations were detected in the baseline plasma ctDNA from 13/14 patients with metastasized disease (Table 2). Pre-treatment tumor DNA available for 12 of these patients tested positive using the drop-off ddPCR assay. Plasma from one patient (patient 6) with metastasized disease had no detectable mutant ctDNA while a *KIT* mutation was detected in the pre-treatment tumor biopsy. In plasma ctDNA collected before start of treatment in eight patients with localized disease and a tumor *KIT* exon 11 mutation, only one patient (sample 7) had a detectable mutation in the ctDNA (Table 3).

**Table 3:** Plasma ctDNA analysis of GIST patients with localized/locally advanced disease

Patient	Primary GIST location	Disease status	Mutation		Mutant allelic frequency
7	Rectum	Localized	KIT exon 11	c.1676T>A	1,95%
9	Stomach	Localized	KIT exon 11	c.1671_1672delinsTG	0,00%
10	Stomach	Localized	KIT exon 11	c.1669_1674del	0,00%
12	Stomach	post-surgery	KIT exon 11	c.1673_1717del	0,00%
13	Stomach	Localized	KIT exon 11	c.1662_1674delinsGGAAGAA;	0,00%
17	Stomach	Localized	KIT exon 11	c.1669_1674del	0,00%
19	Small bowel	Localized	KIT exon 11	c.1669T>C	0,00%
37	Stomach	Localized	KIT exon 11	c.1679 T>A	0,00%

Primary tumors of 7 of these patients were tested positive with the assay (patient 7–19 see Table 1, patient 37 data not shown, tumor of patient 38 was not positive). Samples were taken before start of any treatment.

To exclude that the lack of detectable ctDNA mutations was due to low sensitivity of the drop-off ddPCR assay, tumor and plasma samples collected from two patients with metastasized disease (patient 3 and 15) during treatment with a TKI were also tested with a specific ddPCR mutation assay. As shown in Table 4, the mutant fractional abundance is comparable between the ddPCR and the specific assay. In addition, plasma samples from three different patients were tested using the highly sensitive analytical L-PCR technique for the detection of specific *KIT* exon 11 mutations earlier described [22] (Table 5). For this analysis, we selected eight plasma samples from three patients with different *KIT* exon 11 mutations tested with the drop-off ddPCR assay. Low level mutant frequencies detected with the L-PCR technique (<0.1%) were also positive in the drop-off samples. Of the samples that tested negative using the drop-off assay, 4/5 were also negative using the L-PCR technique.

**Table 4:** Correlation between the ddPCR with a mutation-specific assay and the drop-off assay

Patient	Type	Fractional abundance drop-off probe	Fractional abundance mutant specific probe	Mutation
3	Tumor	53%	48%	c.1727_1729del
3A	Plasma	12,02%	11,60%	c.1727_1729del
3B	Plasma	8,70%	7,20%	c.1727_1729del
3C	Plasma	0,70%	0,72%	c.1727_1729del
15	Tumor	35%	33%	c.1676T>A
15A	Plasma	0,90%	1,02%	c.1676T>A
15B	Plasma	5,50%	4,90%	c.1676T>A
15C	Plasma	0,00%	0,00%	c.1676T>A

Two tumor samples and six plasma samples of two patients with metastasized disease were tested with a probe specifically designed for the mutation. ^*^A = before start of treatment, ^*^B = after two weeks of treatment, ^*^C = after 6 weeks of treatment.

**Table 5:** Comparison of L-PCR with ddPCR

Patient	Mutation	L-PCR Mutation/wild type %	ddPCR Mutation/wild type %	Disease status		
7A	c.1676T>A	0,0019	1,95	Localized		Before start of treatment
7B	c.1676T>A	0,0024	0	Localized		1 week treatment imatinib
7C	c.1676T>A	0	0	Localized		4 week treatment imatinib
10A	c.1669_1674del	0	0	Localized		Before surgical treatment
10B	c.1669_1674del	0	0	Localized		3 days after surgery
15A	c.1676T>A	0,0015	0,94	Metastasized		Before start of treatment
15B	c.1676T>A	0,0012	5,60	Metastasized		2 weeks treatment with imatinib
15C	c.1676T>A	0	0	Metastasized		6 weeks treatment with imatinib

To evaluate the sensitivity of our assay, multiple samples of three patients were analysed with the earlier described L-PCR technique. Quantitative L-PCR analysis was performed on 1 ml plasma as reported previously [Maier *et al.*, 2013]. Four samples were scored low-level positive (<0,1% mutant/wild type ratio). When looked at positive/negative samples the results where –except for sample 7B- comparable with the ddPCR assay.

### The detection of mutations in plasma ctDNA at different time points during treatment

**Figure 2:**
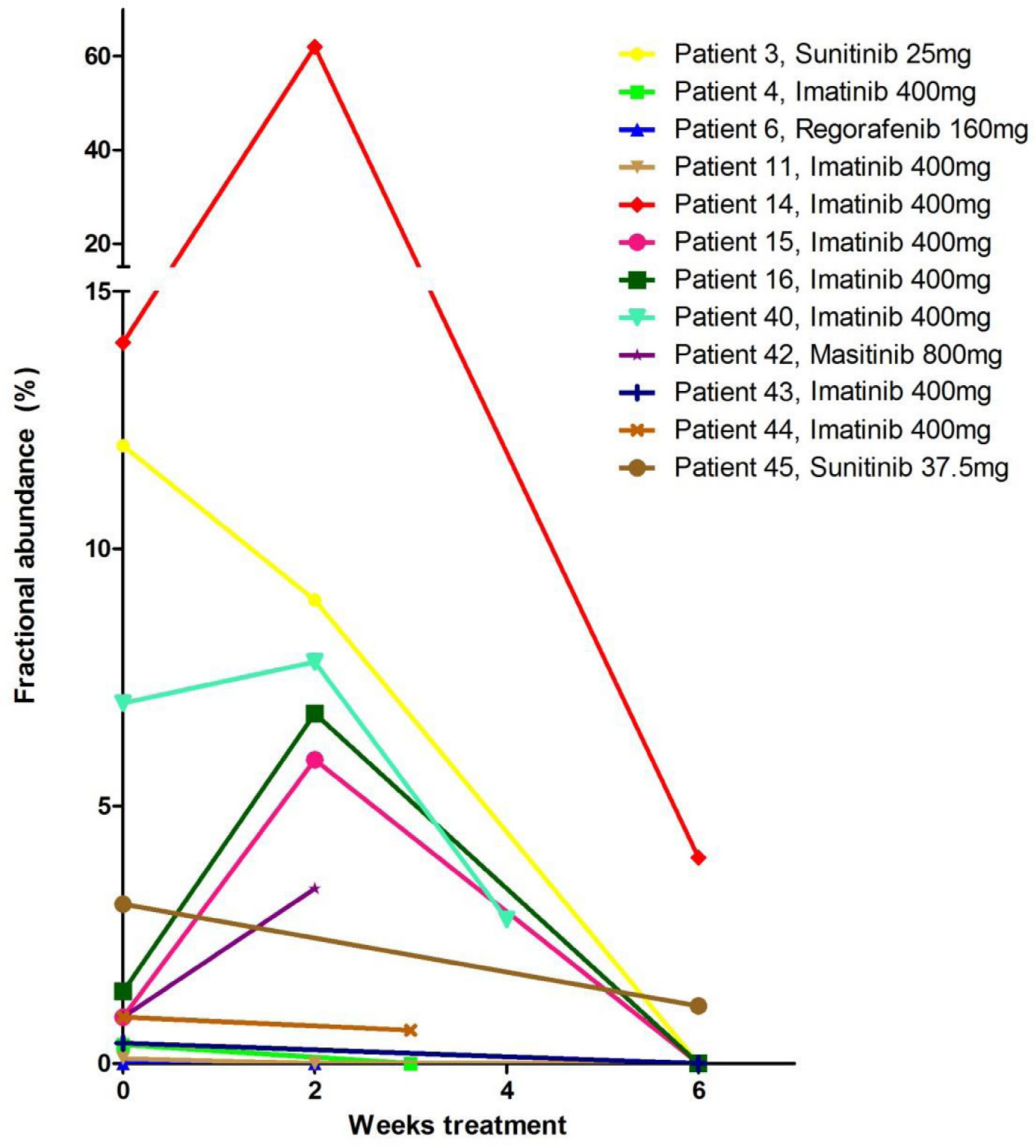
Detection of KIT exon 11 mutations using the ddPCR drop-off assay in ctDNA in patients with metastasized GIST at baseline (before start TKI-treatment) and 2–6 weeks after start of treatment Mutation frequency is expressed as fractional abundance in % (see Supplementary Table 1). Twelve patients with metastasized GIST with both a baseline plasma sample as well as at least one sample collected 2–6 weeks after staring TKI treatment were selected. Both pre-treatment FFPE DNA (Table 1) and baseline plasma samples (Table 2) were tested with the same ddPCR. Patient 39 and 41 (Table 3) were not included since no follow-up plasma samples were available.

In order to monitor the presence of mutations after start of TKI treatment compared to baseline samples, serial plasma samples of 11 patients with metastatic disease using the ddPCR drop-off assay were available and analyzed. The analysis of plasma samples at 2–3 weeks after start therapy revealed an increase in fractional abundance in 5 out of 11 patients (Figure 2, Supplementary Table 1). In all available plasma samples obtained six weeks after start of treatment the fractional abundance decreased below the levels observed at baseline or the 2–3 weeks after start of treatment sample. In agreement with the loss of detection of mutant DNA, ten patients showed a tumor response and one patient stable disease (no. 45) on treatment with TKI according to the first radiological evaluation performed approximately 3 months after start of therapy.

## DISCUSSION

In this study, an in-house designed single ddPCR assay was able to detect multiple mutations in *KIT* exon 11 with high sensitivity (95%) and specificity (100%) in tumor biopsies of patients with GIST. Sensitivity of the assay for all known KIT exon 11 mutations in GIST is lower than 95% since the designed assay covers 80% of the described KIT exon 11 mutations (in the described cohort 21/27 mutations were detected resulting in a sensitivity of 77%). For LOB analysis 5 plasma samples from healthy individuals and 5 normal FFPE samples were analyzed. No false positive droplets were detected in the ddPCR analysis of these samples. As expected, due to the quality of the FFPE material, highly damaged DNA as well as artifactual C>T transitions the ddPCR resulted in a reduced separation of wild type droplets [29]. This should be taken into account when interpreting test results. However, for mutation screening in freshly-processed cell-free plasma DNA, the separation between drop-off and wild type droplets was excellent in all samples tested in this study. Despite a very good LOB and a high sensitivity of 0.1%, the maximum sensitivity that can be obtained is limited by the input of the total number of copies of a genome. As in plasma samples the total amount of DNA is often close to 2ng, this input of DNA would result in a sensitivity of 1%.

This assay enabled the detection of low-level copy mutations and the identification of mutations in 12 of 13 cell-free plasma samples of patients with metastatic disease at baseline. DdPCR is relatively cheap and has a short turn-around time. Since the probe does not detect specific mutations in exon 11 of the *KIT* gene, the drop-off ddPCR assay is especially suitable for predictive testing of GIST in case not enough tissue or neoplastic cells are available for NGS analysis or for monitoring treatment response in ctDNA.

Mutation testing in ctDNA might be an alternative source for tissue biopsies particular when no biopsies or biopsies with insufficient neoplastic cells percentages for molecular profiling are available [30]. In addition, mutation analysis of ctDNA during treatment has been reported as a new tool for monitoring treatment response since the amount of ctDNA correlates with the volume of vital tumor tissue [31]. Circulating DNA in the cell-free plasma fraction originates from many different cells including lymphocytes and neoplastic cells [32]. Their nuclear and mitochondrial DNA is released into the circulation in the process of cellular destruction by apoptosis or necrosis [25]. Therefore, ctDNA in cell-free plasma is a very low fraction of the total amount of circulating DNA. For the detection of mutations in ctDNA in a high background of total plasma DNA, various detection assays with high analytical sensitivity have been reported including digital PCR, BEAMing, sequencing based methods, Ligand PCR, ARMS-PCR and PNA-clamping PCR [33]. Because the analytical sensitivity of NGS is around 1–5% and also requires high amounts of input DNA, NGS is at present not suitable for mutation screening in ctDNA from plasma in malignancies with low abundance of ctDNA. On the other hand, the ddPCR has been reported as a quantitative, accurate assay with high analytical sensitivity [34]. Sensitivities of 0.005–0.1% for *EGFR-T790M* (own unreported data, [28]), 0,1% and 0,5% for *ALK-C1156Y* and *ALK-G1269A* in lung cancer [35] and 0.025% for *KRAS* in CRC [36] are reached.

To the best of our knowledge, this is the first time a single ddPCR assay to detect multiple *KIT* exon 11 mutations in tumor tissue and ctDNA of patients with GIST has been reported. Few other studies have described the use of mutational analysis of ctDNA in GIST. In a recent study, using a NGS platform after enrichment PCR with PNA probes, *KIT* mutations were detected in the plasma of 13 out of 18 patients with localized gastric GIST [37]. With the allele specific ligation PCR assay *KIT* mutations were found in 9 out of 18 patients with active disease, furthermore mutations at low levels were detected in 6 out of 20 patients in complete remission [22]. Another study using BEAMing detected primary mutations in 5 out of 30 patients with TKI-refractory GIST (17%) [23]. Both BEAMing and the allele specific ligation PCR assay require the generation of specific primers/probes for each genomic *KIT* mutation. In GIST patients with localized disease and proven *KIT* mutations in the pre-treatment biopsy, our assay detected the mutations in the baseline plasma DNA in only 1 of 8 cases. An explanation for this discrepancy is that localized tumors may not actively shed tumor DNA into the circulation. In other malignancies an association was reported with the detection of mutations in plasma and advanced stage disease [38].

The ddPCR drop-off assay was previously described for the detection of various clinical-relevant deletions in exon 19 of the *EGFR* gene in lung cancer [28]. This ddPCR drop-off del 19 assay showed a sensitivity of 5–50 mutant copies in a background of 10,000 wild type copies which is similar to our observed sensitivity for the ddPCR drop-off assay for *KIT* exon 11 mutations.

In the analysis of serial ctDNA samples, an evident rise of fractional abundance was seen after initiation of treatment. We hypothesized that the rise of mutational level could be due to increased cell death induced therapy initiation. This early response is not reported in other malignancies treated with TKI [39]. Our result implies that treatment response can be monitored by using this ddPCR assay in cell-free plasma. Similar observations were also reported using quantitative L-PCR in 5 patients with advanced GIST [22]. Monitoring of treatment response has also been reported in anti-*EGFR* treated CRC using *KRAS* mutations [40], TKI-treated lung cancer for *EGFR del19/L858R* [41], *BRAF* mutated melanoma [42] and gynecologic malignancies [43] and detection of progression on primary TKI in ctDNA has been reported in *EGFR* mutated NSCLC [44] and CRC [45].

Since tumors evolve during treatment and secondary mutations can cause therapeutic resistance, a new biopsy can be required during treatment to define the actual mutational status [46]. This has recently been demonstrated in patients with NSCLC during treatment with *EGFR*-TKI. The *EGFR* TKI-resistance mutation *T790M* was detected in ∼70% of plasma ctDNA of patients with advanced disease who had acquired TKI-resistance [47]. These resistant mutations could be missed by conventional tissue biopsy due to tumor heterogeneity [48]. In addition, repeated tumor biopsies have risks e.g. bleeding, perforation and infection. Thus, there is a need for less invasive techniques that provide information about mutational status of tumors and that can be easily performed at different time points during treatment. The detection of primary and secondary resistant mutations in ctDNA cannot be used only to monitor recurrences before clinical manifestation, but might also warrant a different therapeutic approach. Recently, the FDA approved the detection of the *EGFR* TKI resistant *T790M* mutation in plasma (June 1, 2016) as a marker for a second generation *EGFR* TKI specifically inhibiting the *T790M* mutation [49]. Similarly, in GIST, resistance develops during imatinib treatment. In 50% of patients with progressive disease, a secondary mutation, besides the primary *KIT* mutation, is detected [50]. Treatment response to standard second line therapy, sunitinib, differs between patients with secondary *KIT* exon 13/14 or exon 17/18 mutations [51]. The detection of secondary mutations in plasma was reported in 4 patients using pre-amplification and NGS. Mutant alleles were detected in a range of 0.010–9.385% [24]. In a study using BEAMing secondary mutations were detected in 11 out of 30 patients (41%) [23]. Therefore, the implementation of ddPCR (or other sensitive) detection assays to identify resistant *KIT* mutations in plasma ctDNA is warranted for the development of more optimal treatment strategies in patients with GIST treated with TKIs.

The detection of multiple *KIT* exon 11 mutations with a single ddPCR assay has high sensitivity and specificity. It is suitable for predictive testing of GIST in case not enough tissue or neoplastic cells are available for routine NGS analysis in FFPE tissue. This technique can be easily performed, is cost-effective and has a short turn-around-time. Therefore, this ddPCR assay might be especially suitable for treatment response monitoring by ctDNA analysis in plasma. Our study will be extended to include the monitoring of early progression based on ctDNA, which may guide early treatment adaptations.

## MATERIALS AND METHODS

### Study design

The reported work is part of an open-label, non-randomized, non-interventional, explorative multicenter study aiming to detect the most frequently occurring *KIT* exon 11 mutations using a single ddPCR assay. The assay was first tested on archival formalin-fixed paraffin-embedded (FFPE) tumor tissue stored at the University Medical Centre Groningen (UMCG). After validation with tumor tissue, this assay is tested in prospectively collected plasma samples from 22 GIST patients before and during treatment with a TKI. These 22 patients were treated in one of the five hospitals in the Dutch GIST consortium (Antoni van Leeuwenhoek, Amsterdam; Leiden University Medical Centre, Leiden; Erasmus University Medical Centre, Rotterdam; Radboud University Medical Centre, Nijmegen; University Medical Centre Groningen, Groningen). 405 plasma samples of 140 GIST patients before or during treatment with a TKI have been prospectively collected (Dec 2014 - Sept 2016). Treatment, follow-up and response evaluation by CT according to RECIST 1.1, were performed according to (inter)national guidelines. Plasma samples were available before start and at multiple time points after start of a TKI for 8 patients with localized GIST and 14 patients with metastasized GIST. All patients had measurable disease before collection of the first plasma sample and received systemic treatment during the study period. Plasma samples were collected at every visit to the outpatient clinic. Disease evaluation was performed by CT-scans performed approximately every 3 months. Response evaluation was performed using RECIST version 1.1 criteria by a radiologist, unaware of obtained ctDNA results.

All patients gave written informed consent. The Medical Ethical Committee approved the study and it is registered on ClinicalTrials.gov (NCT02331914).

### Tumor sample collection and DNA extraction

Pre-treatment FFPE tumor biopsies for 27 consecutive patients diagnosed with exon 11 mutated GIST between 2012 and 2015 were retrieved from the local pathology archive at the UMCG . Nine GIST tumors with mutations outside *KIT* exon 11 from the same period were randomly selected as controls. FFPE samples of healthy controls were obtained from the pathology department of UMCG. Tumor-specific mutations were determined by routine diagnostic NGS of a gene panel with relevant predictive markers (version PGMv001; www.moloncopath.nl) on the IonTorrent platform (Thermo Fisher Scientific, Waltham, MA, USA). The analysis of some older tumors was performed using Sanger sequencing as reported previously [52].

In brief, two to four 10 µm thick sections were cut from the original FFPE blocks preceded and followed by a 4 µm section. After haematoxylin and eosin staining, the 4 µm slides were evaluated by an experienced pathologist for the presence of an area with sufficient tumor cells (>20%). Genomic DNA from FFPE slides was extracted using the Cobas extraction kit (Roche, Basel, Switzerland) and quantified using Qubit (Thermo Fisher Scientific). All molecular testing was performed in the CCKL/ISO15189-accredited laboratory of molecular pathology at the UMCG. All standard precautions were taken to avoid contamination of amplification products using separate laboratories for pre- and post-PCR handling. To avoid cross-contamination, a new microtome blade was used each time a new sample was sectioned.

### Next generation sequencing using IonTorrent

Libraries were generated using an in-house panel (version PGMv001) using the IonTorrent platform. This panel consists of 30 primer pairs covering 11 clinically relevant genes including hotspots in exon 9, 11, 13 and 17 of *KIT* and exon 12, 14 and 18 of *PDGFR*α (http://www.moloncopath.nl). 10 ng of DNA from each sample was used to prepare barcoded libraries using IonXpress barcoded adapters (Thermo Fisher Scientific). Libraries were combined to a final concentration of 100 pmol using the Ion Library Quantification Kit (Thermo Fisher Scientific), and emulsion PCR was performed using the IonTorrent OneTouch TM2 system. Samples were sequenced on the IonTorrent semi-conductor sequencer using Ion 316 or 318 chips. Sequence reads were aligned to the 11 genes based on the Human Genome version 19 using Sequence Pilot v4.2.0 (JSI Medical Systems GmbH, Ettenheim, Germany). Also read depth and uniformity of coverage across individual amplicons were assessed. In data analysis the cut-off was set at mutations found in > 5% of the reads. Only non-synonymous and non-sense variations in coding regions were included.

### Drop-off ddPCR assay

**Figure 3:**
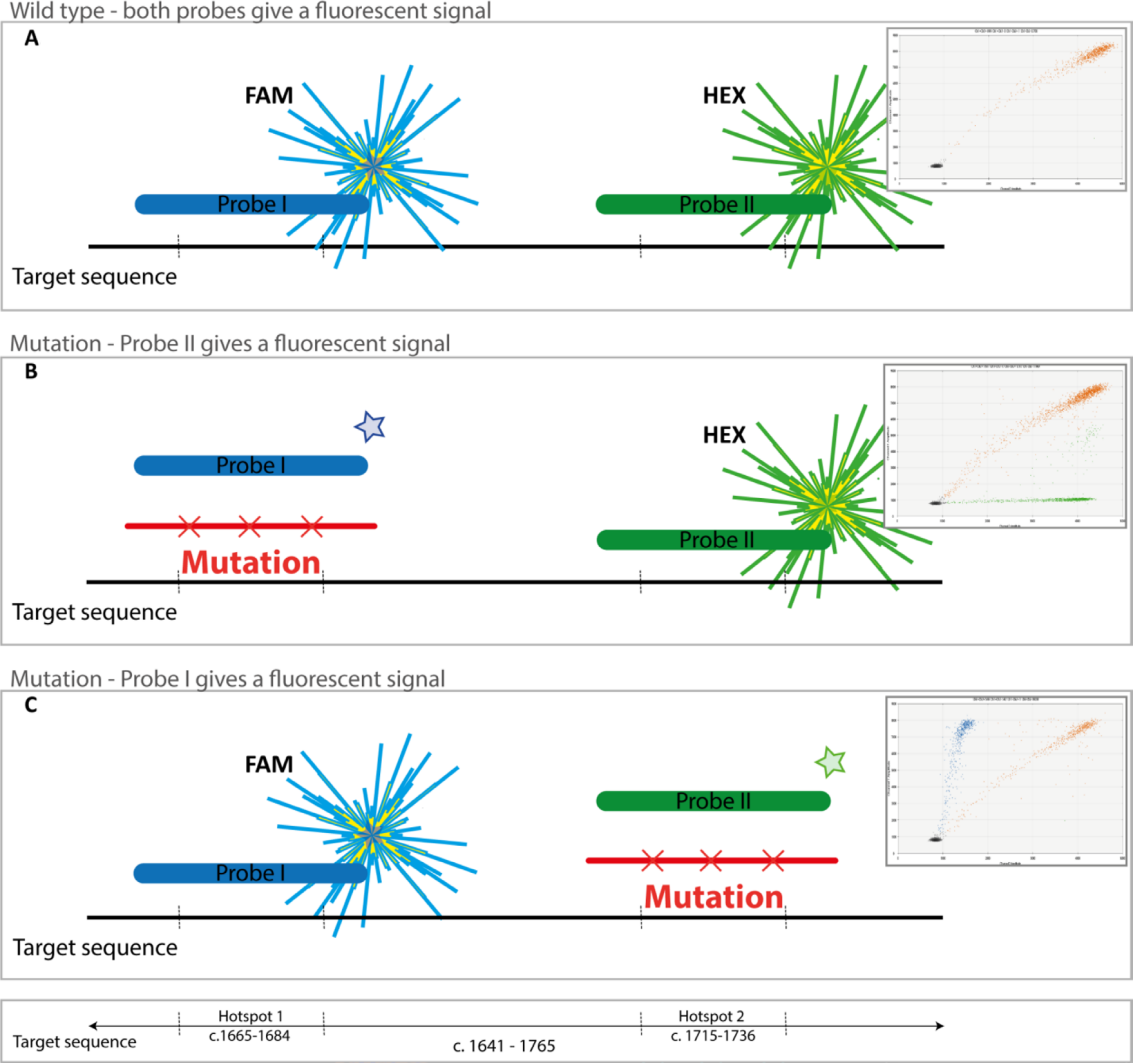
KIT exon 11 mutation/deletion detection assay (**A)** When no mutation is present, both probes (FAM and HEX) will anneal and droplets with a dual fluorescent signal will be detected (coloured orange in the figure). (**B)** In cases with a mutation in hotspot region I, only droplets with WT region II are detected (HEX, green signal). Also wild type fragments are detected (orange droplets) in the graph. (**C**) Example of a case with a mutation in hotspot region II, only droplets with WT region I are detected (FAM, blue signal). Wild type fragments are also detected (orange droplets).

Since in 80% of the cases exon 11 mutations occur in one of the two hotspot regions, one probe serves as a wild type probe while the loss of signal from the second probe represents the presence of a mutation (Figure 3). A ddPCR assay consisting of a single set of PCR primers and two TaqMan probes (FAM or HEX) was designed using PrimerQuest (http://eu.idtdna.com/Primerquest) and purchased from IDT (Coralville, IA, USA). The primer sequences are Fwd. 5′-CCACAGAAACCCATGTATGAAG-3′ (position c.1641-c.1662) and Rev. 5′-GAGTTTCCC AGAAACAGGC-3′ (position c.1746-c.1765) resulting in a PCR product of 124 base pairs covering both hotspots in *KIT* exon 11 (position c.1641-c.1765, Figure 3). The sequence of probe I (FAM) is 5′-ACAGT GGAAGGTTGTTGAGGAGAT-3′ and probe II (HEX) 5′-ACCCAACACAACTTCCTTATGATCACA-3′. Temperature gradient PCRs of the primers and probes were performed to detect the optimal annealing temperature and resulted in an optimal PCR temperature of 60°C.

### Specific ddPCR assays

For the detection of the c.1676T>A mutation, a commercially available assay was purchased (dHsaCP2506828 and dHsaCP2506829, Bio-Rad, Hercules, CA, USA). The specific c.1727_1729del assay was designed in-house and purchased from IDT. The primer sequences are Fwd. 5-′CCACAGAAACCCATGTATG-3′ (position c.1643-c.1661) and Rev. 5′-GCCTGTTTC TGGGAAAC-3′ (position c.1750-c.1766). The sequence of wild type-probe I (FAM) is 5′-ACCCAACACAACCTTATGATCACAAATG-3′ and mutant-probe II (HEX) 5′-ACAGTGGAAGGTTGTTGAGGAG-3′.

### DdPCR analysis of DNA of pretreatment tumor biopsies

DdPCR on tumor tissue was performed on 2 ng of genomic DNA as measured by Qubit according to the manufacturer’s instructions. Briefly, 11 µl ddPCR Supermix for probes, 1 µl of the ddPCR assay (wild type and mutation primer/probes) and genomic DNA were mixed in a final volume of 22 μl. Droplets were generated from 20 μl of the suspension using the QX100 Droplet generator after addition of 70 μl droplet generation oil (Bio-Rad). The PCR was performed on a T100 Thermal Cycler (Bio-Rad) using the following cycling conditions: 10 minutes at 95° C, 40 cycles of 95° C for 30 seconds, 60° C for 1 minute followed by 98° C for 10 minutes (ramp rate 2.5° C/sec). Samples were transferred to the QX200 Droplet Reader (Bio-Rad) for fluorescent measurement of FAM and HEX probes and data were analyzed using Quantasoft software version 1.6.6. Samples were considered positive when 3 or more FAM/HEX positive droplets were detected, while no FAM/HEX positive droplets in the no-template and no single positive droplets in the wild type controls were observed. The fractional abundance is based on the ratio between mutant and wild type droplets after correction using the Poisson distribution (calculated by the Quantasoft software).

### Circulating tumor DNA analysis

Plasma samples from patients treated at the UMCG were collected in EDTA tubes (vacutainer #367525, Becton Dickinson, Franklin Lakes, NJ, USA) and processed within 4 hours after venipuncture. Samples from patients from other centers were collected in cell free DNA BCT tubes (Streck, Omaha, NE, USA), which stabilizes blood samples for a minimum of 7 days at room temperature [53]. The cell free BCT tubes were sent by regular mail to the UMCG and processed on the day of arrival. For quantitative validation of the assay, plasma samples of five anonymous healthy controls were collected in the same cell free BCT tubes.

EDTA samples were first centrifuged for 10 minutes at 820 *g* to separate the lymphocytes from the plasma. The supernatant was transferred to a new Eppendorf tube and centrifuged at 16,000 *g* for 10 minutes to separate plasma from the remaining debris. After the last centrifugation step the supernatant was transferred and stored at −80° C until analysis. Cell free DNA BCT tubes were processed identically but with a different first centrifugation step (1,600 *g*). Plasma processing was performed in a laboratory not used for any molecular testing.

For ctDNA isolation, samples were thawed after storage at –80° C and centrifuged for 5 minutes at 16,000 *g*. DNA was extracted from plasma using the QIAamp Circulating Nucleic Acid kit. (QIAgen, Hilden, Germany) following manufacturers protocols. DNA from 4 ml of plasma was isolated and eluted in 2 × 250 µl of elution buffer. This eluate was concentrated using an Amicon filter column (Merck, Darmstadt, Germany). The final amount of eluate was 15–20 µl. After isolation the eluate was stored at 4° C until experiments were performed.

The designed drop-off ddPCR assay was used for analysis of ctDNA. Experimental conditions were identical to those for analysis of tumor tissue except for the input per reaction. For analysis of ctDNA the maximum input (9 µl) of isolated DNA per reaction was used, each run included wild type (WT) and no template controls (NTC). The presence of a mutation was calculated as the fractional abundance.

Quantitative L-PCR using 1 ml of cell-free plasma was performed as reported in detail previously [22]. The laboratory technicians who performed the ddPCR experiments were not aware of the mutational and clinical status of the tested patient samples.

### Quantitative performance of the drop-off ddPCR assay

The sensitivity of the assay was determined using DNA from FFPE pretreatment biopsies with mutations in exon 11 hotspot 1 (c.1669T>C and c. 1671_1676del) and hotspot 2 (c.1727_1729del) with known mutation allelic frequency (MAF) determined by NGS diluted with wild type DNA. A significant correlation was observed between tumor DNA input as measured by NGS-MAF and mutated droplet detection in three different samples (Supplementary Figure 4). The limit of detection (LOD) of the drop-off ddPCR assay on DNA extracted from FFPE-tissue with 30 ng DNA input was 0.11% (not shown) and with a lower DNA input (2 ng) a maximum of 1% mutant alleles was still detected (see example in Supplementary Figure 5).

The limit of blank (LOB, false mutation rate) as reported earlier [54] of the ddPCR assays was estimated using five FFPE healthy tissue. FFPE samples were tested with an input of 2ng and 30ng resulting in respectively in a mean of 545 and 5345 wild type droplets and 0 false-positive droplets per sample (LOB = 0% for tissue DNA). To determine the LOB on plasma-derived ctDNA, cell free DNA was isolated from five plasma samples of healthy controls following the study extraction protocol and maximum input was used resulting in a mean of 791 wild type droplets and 0 false-positive droplets (LOB = 0% for normal plasma DNA, Supplementary Figure 6).

## SUPPLEMENTARY MATERIALS FIGURES AND TABLE


