# Supplementary material for: A single digital droplet PCR assay to detect multiple KIT exon 11 mutations in tumor and plasma from patients with gastrointestinal stromal tumors
Source: Oncotarget. 2018 Feb 14;9(17):13870–83. doi: 10.18632/oncotarget.24493 (PMC5862622; doi:10.18632/oncotarget.24493)
Supplement: Supplementary file 1 [file oncotarget-09-13870-s001.pdf]

A single digital droplet PCR assay to detect multiple *KIT* exon 11 mutations in tumor and plasma from patients with gastrointestinal stromal tumors

SUPPLEMENTARY MATERIALS

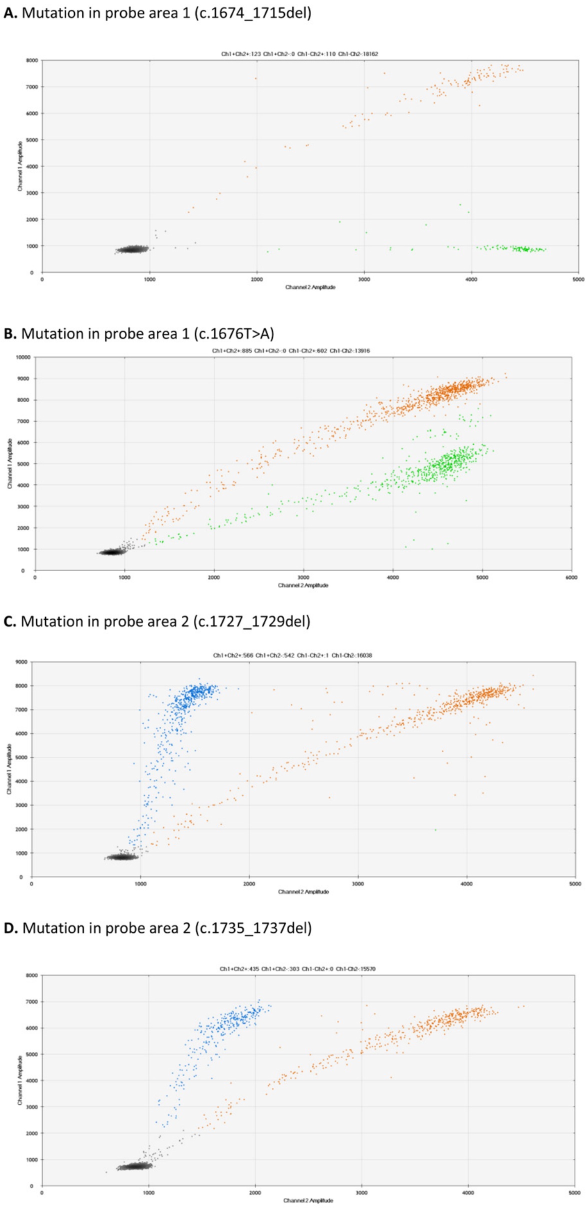

Supplementary Figure 1: Examples of ddPCR drop-off results of FFPE-samples with a KIT exon 11 mutation. (**A**) Tumour 24 contains a deletion in probe area 1. It shows a common pattern observed in most cases with mutant (green) and wild-type (orange) droplets. (**B**) Tumour 7 with a point mutation in area 1 with mutant (green) and wild-type (orange) droplets. (**C**) Tumour 6 with mutation in the second probe area shows mutant droplets (blue) and wild-type droplets (orange). (**D**) Tumour 23, with deletion in the second probe area, shows mutant droplets (blue) and wild-type droplets (orange).

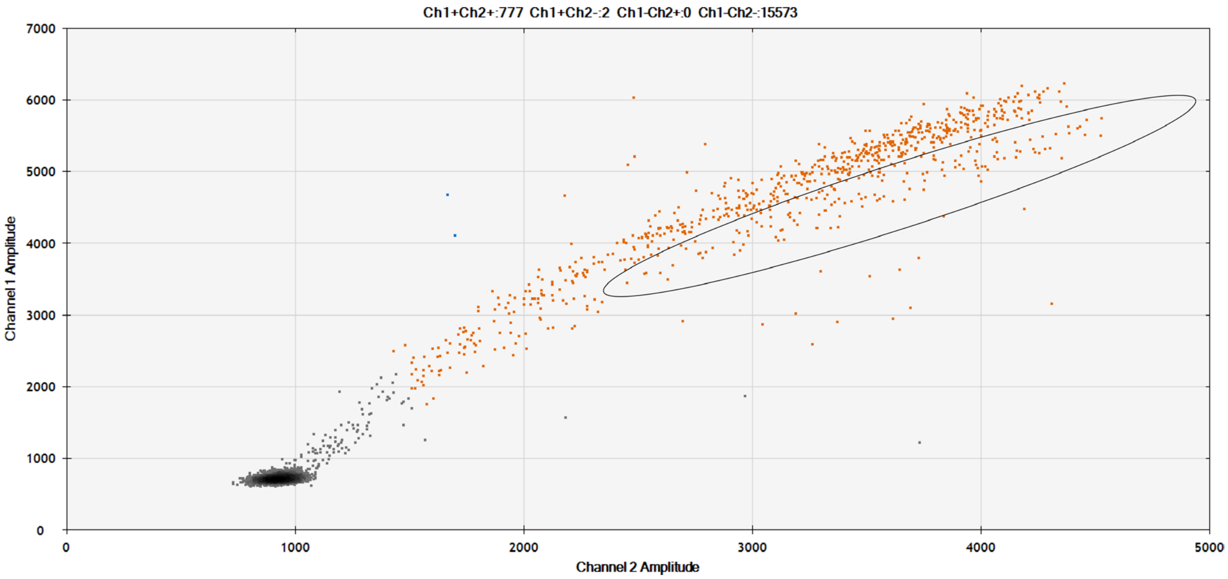

Supplementary Figure 2: Tumor 18 carries a duplication (c.1719_1751dup). The ddPCR drop-off assay did not result in a common drop-off pattern seen in other cases with mutations and deletions (see Supplementary Figure 1). However, within the wild-type cluster region defined by the orange droplets, in addition to the prominent wild-type cluster, a separate cluster with slightly lower fluorescence intensity can be distinguished marked by the black oval. The number of signals of these clusters is similar to the mutant allelic frequency of 11% as determined with NGS on the same DNA and strongly suggests that this cluster represents the mutant droplets.

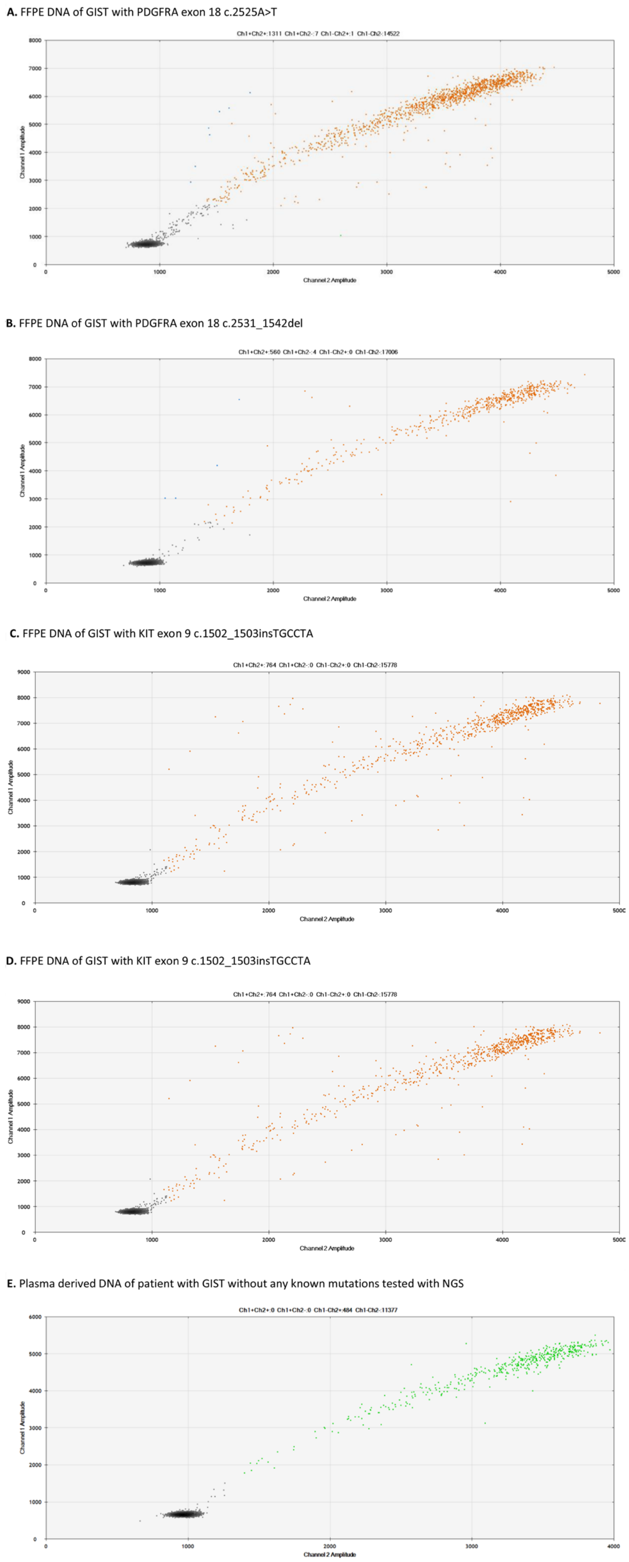

Supplementary Figure 3: Examples of tumours without KIT exon 11 mutations and considered as negative controls. (**A**–**D**) The ddPCR drop-off assay was performed on 2 ng input DNA extracted from FFPE tissue of GIST with mutations other than KIT exon 11. (**E**) DdPCR analysis of plasma derived DNA (input 10 ng). According to our criteria these samples are considered negative although few scattered positive droplets were observed outside the clusters commonly detected for HEX only, FAM only or double HEX/FAM droplets (see Supplementary Figure 6). Using fluorescent measurement of FAM and HEX probes and data analysis with Quantasoft software version 1.6.6, in general we define only droplets above channel amplitude 1000 for HEX and above 2000 for FAM as true positive. The amount of these scattered droplets is associated with the quality of DNA and therefore sometime observed in DNA from old-FFPE tissue blocks and very rarely in plasma derived DNA.

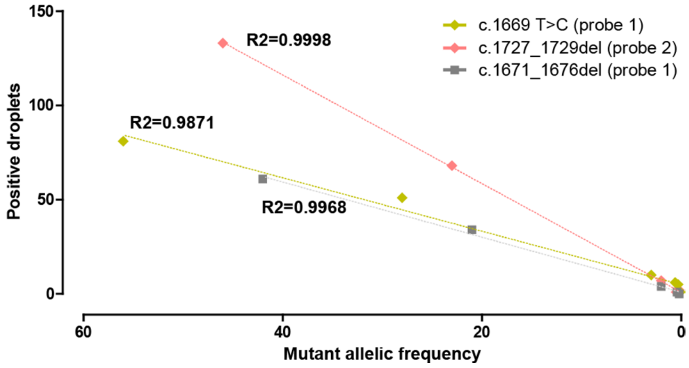

Supplementary Figure 4: Correlation between mutant allelic frequencies as determined with NGS and positive droplets detected by ddPCR of three different tumour FFPE samples. DNA input is 2 ng. Two samples have a KIT exon 11 mutation in hotspot 1 and one sample a mutation in hotspot 2.

A. FFPE DNA with 100% neoplastic cells (undiluted) with a KIT c.1671_1676del mutation.

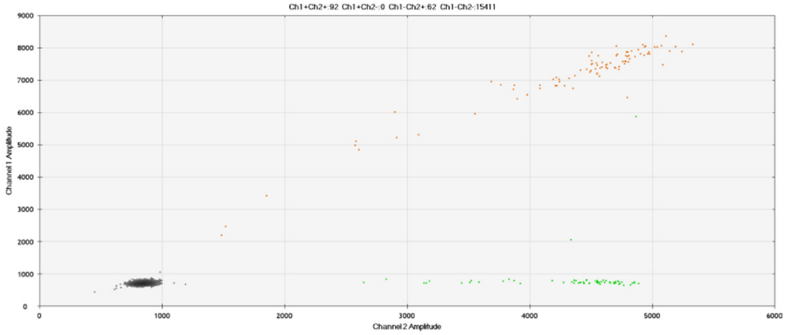

B. FFPE DNA with 50% neoplastic cells with a KIT c.1671_1676del mutation.

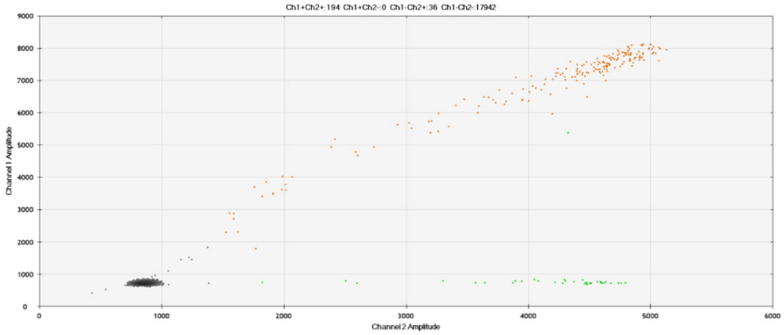

C. FFPE DNA with 5% neoplastic cells with a KIT c.1671_1676del mutation.

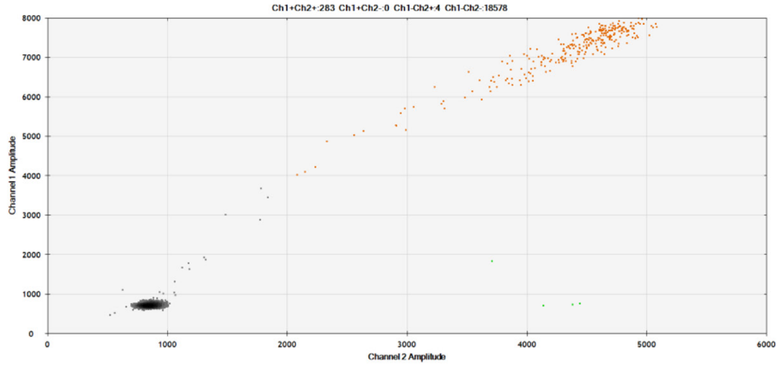

D. FFPE DNA with 1% neoplastic cells with a KIT c.1671_1676del mutation.

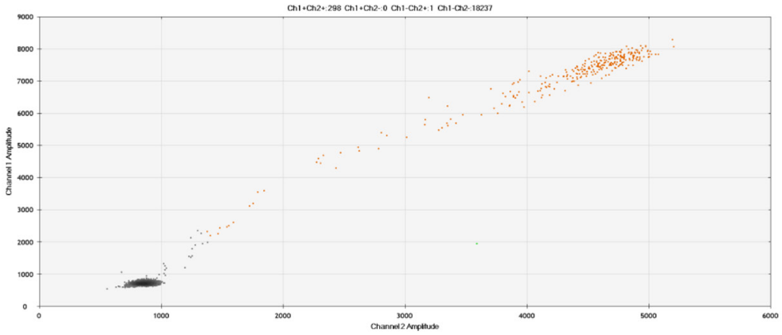

Supplementary Figure 5: DNA dilution series of a FFPE tumour sample with a KITc.1671_1676del mutation using 2 ng total DNA input. (**A**) ddPCR analysis of undiluted tumour DNA (100% neoplastic cells) detected 61 positive droplets and a fractional abundance of 40%. NGS revealed a similar mutant allelic frequency of 42%. (**B**) ddPCR of 50% dilution results in 34 positive droplets and a fractional abundance of 16%. (**C**) ddPCR of 5% tumour dilution results in 4 positive droplets and fractional abundance of 1%. (**D**) ddPCR of 1% tumour dilution resulted in 1 detectable droplet according to our criteria considered as negative (no deletion/mutation is present).

A. DdPCR of plasma sample from healthy donor, input is 9.72ng.

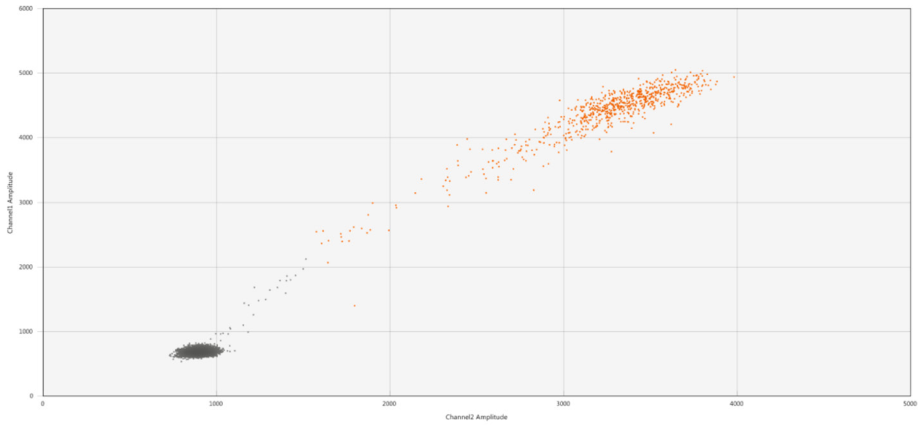

B. FFPE sample of a tonsil, input of 2ng.

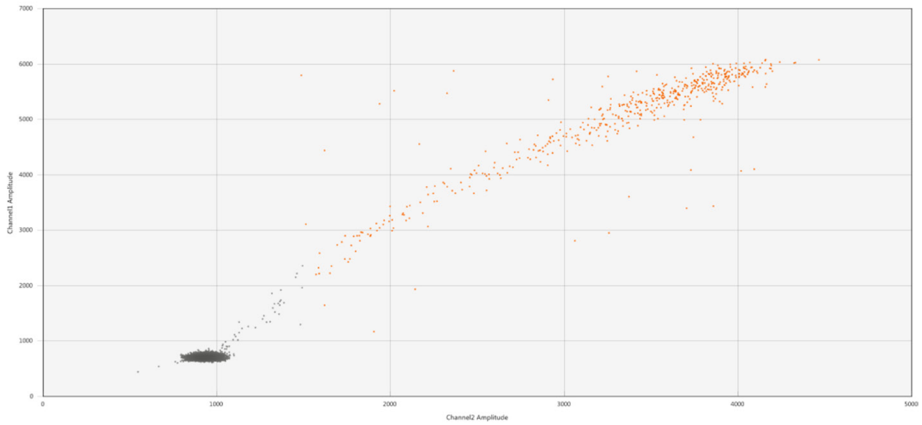

C. ddPCR of same FFPE sample as S6B but with input of 30ng.

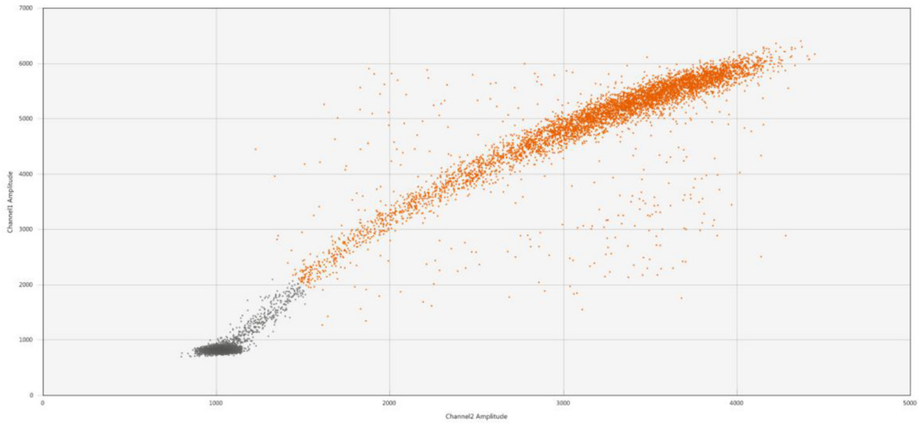

Supplementary Figure 6: LOB detection of FFPE and plasma samples. (**A**) Plasma sample of a healthy donor, input is 9.72 ng. (**B**) ddPCR of a FFPE tonsil sample with 2 ng input, a broader range of wild type is detected compared to the plasma sample. (**C**) Same FFPE tonsil sample as S6B with input of 30 ng. A broader range on both sides of the wild type droplets are detected but none were positive for a single probe considered as drop-off (also see legend in Supplementary Figure 3).

Supplementary Table 1: Fractional abundance of mutant alleles at baseline and after 2–3 weeks and 4–6 weeks after start of treatment (also see Figure 2)

Patient	Fractional abundance at baseline	Fractional abundance at 2–3 weeks treatment	Fractional abundance at 4–6 six weeks treatment
3	12,0%	9,0%	0,0%
4	0,4%	0,0%	0,0%
6	0,0%	0,0%	–
11	0,1%	0,0%	0,0%
14	14,0%	62,0%	4,0%
15	0,9%	5,9%	0,0%
16	1,4%	6,8%	0,0%
40	7,0%	7,8%	2,8%
42	0,9%	3,4%	-
43	0,4%	0,0%	0,0%
44	0,9%	0,7%	–
45	3,1%	–	1,1%
